# 

**Published:** 1888-04

**Authors:** 


### CONTENTS.

***MISCELLANEOUS.*** PAGE

Psora. S. L. G. Leggett, M. D., . . . . . . . . . . 159
Sycosis. Prof. J. T. Kent, . . . . . . . . . . . 163
The Characteristics of Ten Tissue Remedies. E. J. Lee, M. D.:
Calcarea Fluorica, . . . . . . . . . . 169
Calcarea Phosphor., . . . . . . . . . . 169
Calcarea Sulphurica, . . . . . . . . . . 171
Ferrum Phosphor., . . . . . . . . . . 172
Kali Muriaticum, . . . . . . . . . . 172
Kali Phosphor., . . . . . . . . . . 173
Kali Sulphuricum, . . . . . . . . . . 173
Magnesia Phosphor., . . . . . . . . . . 174
Natrum Phosphor., . . . . . . . . . . 174
Natrum Sulphuricum, . . . . . . . . . . 176
Mercurial Poisoning, . . . . . . . . . . . 178
A Case of Fatal Poisoning by Chininum Sulph. S. L., . . . . 179
Some Clinical Cases. Geo. W. Sherbino, M. D., . . . . . 179
A First Experience. F. M. Gustin, M. D., . . . . . . 182
Clinical Notes. Dr. H. W. Andrews, . . . . . . 184
Fish-brine Odor E. W. Berridge, M. D., . . . . . 185
A Verification of Cicuta Viroso. Mr. Alfred Heath, . . . 186
Some Tributes to the Memory of the Late Dr. Lippe, . . . 186
Uses of Oranges, . . . . . . . . . . . 192
Therapeutics of the Throat: Rhus Toxicodendron. Dr. C. E. Chase, 193
The Syracuse Hahnemannian Club, . . . . . . . . 197
Proceedings of The Hahnemann Club of Toronto, . . . . . 198
Remedies for Post-Partum Hemorrhage: Secale. Dr. J. D Tyrrell, 199
Ustilago. L. Hamilton Evans, M. D., . . . . . . . . 200
Phosphorus. Dr. A. B. Eadie, . . . . . . . . . . 201
Pulsatilla. Dr. Edward Adams, . . . . . . . . . . 202
Cinnamonium. Dr. John Hall, Sr., . . . . . . . . . 203
Sabina. Dr. W. J. Hunter Emory, . . . . . . . . . 204
Proceedings of The Organon Society of Boston, . . . . . 204
Homœopathic Medical College of Missouri, . . . . . . 210

***BOOK NOTICES AND REVIEWS.***

Proceedings of the Twenty-third Annual Meeting of the Homœopathic Medical Society of Ohio, . . . . . . . . . 211
Scientific Medication and Specific Medicines. Dr. John M. Scudder, 211
Fox's Atlas and Text-Book of Skin Diseases, . . . . . . 211
A Materia Medica. Samuel Swan, M. D., . . . . . . . 211

***NOTES AND NOTICES.***

Nebraska State Homœopathic Society—The Hahnemannian Monthly —Lectures on the Organon—Errata—Location Desired—What!! —A Surgical Journal—For Sale, . . . . . . . . 212

